# Key Challenges in Rheumatic and Musculoskeletal Disease Translational Research

**DOI:** 10.1016/j.ebiom.2014.11.011

**Published:** 2014-11-18

**Authors:** Ursula Fearon, Douglas J. Veale

**Affiliations:** St. Vincent's University Hospital, Elm Park, Dublin 4, Ireland

Biotherapeutics have greatly advanced the world of medicine, and products such as monoclonal antibodies, fusion proteins and receptor antagonists have transformed the therapeutic armamentarium for rheumatic and musculoskeletal diseases (RMD) in the last 20 years. Biological understanding of a disease can result in novel targeted therapeutics, whereas new molecular pathological insights can stem from observations when patients have responded differently to the same treatment. Rheumatoid arthritis (RA) provides an excellent example of the bidirectional nature of translational research involving clinicians and scientists working in concert with mutual understanding of both the clinical problem and the scientific solution. RA biotherapeutics are effective in many but not all patients, and adverse effects, namely increased infections, may limit their use. The current challenges for translational research and biomedicine in RMD/RA therefore focus on the following issues: (i) early diagnosis, (ii) personalised medicine, and (iii) assessment of outcome. The unifying theme to these challenges is the development of biomarkers, as will be evidenced in the next paragraphs.

There have been significant advances in relation to early diagnosis for RMD over the past 10 years. Clinical awareness and the importance of an early diagnosis were increased from 1991 with the growth of early arthritis clinics and the application of the 1987 diagnostic criteria for RA. These classification criteria were updated in 2010 with significant changes. A new biomarker for RA – anti-citrullinated antibody (anti-CCP) – was first suggested when citrulline was described as an essential antigenic constituent of RA-specific antibodies (Schellekens et al., 1998). Anti-CCP antibodies only became widely available commercially as recently as 2007, and since this time their utility has been expanded to include both diagnostic and prognostic markers of RA, as they are recognised to be associated with poor prognosis or outcome as well as diagnosis (De Rycke et al., 2004). Even more recently, the presence of anti-CCP and rheumatoid factor antibodies has been described in some subjects year before they develop the signs or symptoms of the disease (Rantapää-Dahlqvist et al., 2003). This knowledge has led to a number of strategic studies exploring treatment options for very early RA, at a stage that subjects with joint symptoms and positive antibodies may receive therapy before signs of RA develop. The full implications of these studies are awaited (Gerlag, 2013).

The concept of personalised medicine is not new, however the rapid advances in technologies such as genomics, transcriptomics and metabolomics for the analysis of blood and tissue has opened up new horizons in this area in the last few years (Isaacs and Ferraccioli, 2011). As outlined above, the range of biotherapeutics now available for the treatment of RMD is extensive, and the key challenge for translational scientists is to create and validate a strategic approach to the treatment of RA (Ma et al., 2014). Early diagnosis is only the first step, subsets of patients need to be stratified by a reliable biomarker, or more likely a combination of biomarkers, that will allow treatment decisions to follow an algorithm defined on a pathological basis, similar to that which has been developed for the treatment of breast cancer (Perou et al., 2000). For example, it has been suggested that B-lymphocyte infiltrates on immunohistochemistry of synovial tissue may be a biomarker of response to therapy with rituximab in RA patients.

In order to gauge the success or failure of treatments in RMD, following early diagnosis and strategic patient stratification, we need reliable and feasible outcome measures. A multidisciplinary group of researchers have attempted to refine and develop Outcome MEasures in Rheumatoid Arthritis Clinical Trials (OMERACT) using a progressive and well-defined methodology, combining literature review and consensus over the past 20 years (Kirkham et al., 2013). Initially, the focus of the group was on RA alone; however over time, they have extended this approach to most RMD and included clinicians, nurses, industry partners and, possibly most importantly, the patients themselves. This group was the first to identify and validate a synovial tissue biomarker – CD68 – to reflect synovial pathologic responses in clinical trials (Smith et al., 2006). It may be relatively easy to define the patient who achieves complete remission – no clinical signs or symptoms of disease activity and no residual damage – but this is unfortunately quite rare. According to the most recent studies examining the new EULAR/ACR remission criteria, complete remission may apply to only 10% or less of treated patients (Balogh et al., 2013).

In conclusion, there are significant challenges ahead for clinicians and scientists engaged in translational research in RMD, and specifically in RA. There is, however, significant potential for advances to be made, in particular using modern technologies applied to blood and synovial tissue samples in subjects with well-defined clinical characteristics combined in a systems biology analysis.

## Conflicts of Interest

The authors declared that there are no conflicts of interest.
